# Caliber-persistent labial artery: report of 3 cases^☆^

**DOI:** 10.1016/j.abd.2021.01.004

**Published:** 2021-11-25

**Authors:** José Antonio Llamas Carmona, Ángela Rivera Mercado, Miguel Lova Navarro, Elisabeth Gómez Moyano

**Affiliations:** a Dermatology Department, Hospital Regional Universitario de Málaga, Málaga, Spain; b Radiology Department, Hospital Regional Universitario de Málaga, Málaga, Spain; c Dermatology Department, Hospital Clínico Universitario Virgen de la Arrixaca, Murcia, Spain

**Keywords:** Biopsy, Bleeding, Lip, Lip diseases, Pulsatile flow, Ultrasonography, Doppler

## Abstract

The caliber-persistent labial artery is a vascular anomaly in which a primary arterial branch penetrates into the submucosal tissue without reduction in diameter. Most lesions are benign and do not require treatment, except for complications and/or on patient demands. In this way, noninvasive diagnostic tools are preferred such as high-resolution and color Doppler ultrasonography which allow direct observation of the lesion, assessing its exact location and diameter at every axis, as well as the blood flow velocity. An excisional biopsy of these lesions or even their surgical extirpation could have a fatal outcome with profuse bleeding.

## Case report

The authors present three further cases of Caliber-Persistent Labial Artery (CPLA) with different clinical presentations each one, diagnosed clinically in the study’s department over a 12-month period using non-invasive diagnostic tools avoiding the biopsy and its complications.

### Case report 1

A 58-year-old male with no previous relevant medical history presented with the complaint of an asymptomatic, erythematous-violaceous, non-ulcerated, soft-tissue, progressively growing tumoration (over the last 8 months) on the left side of the lower lip. The patient refers history of trauma to the lip.

The clinical examination revealed a slightly raised nodule measuring 3 cm pulsatile on palpation. Dermoscopy showed linear and tortuous blood vessels with superficial micro ulcerations.

**Figure 1:**
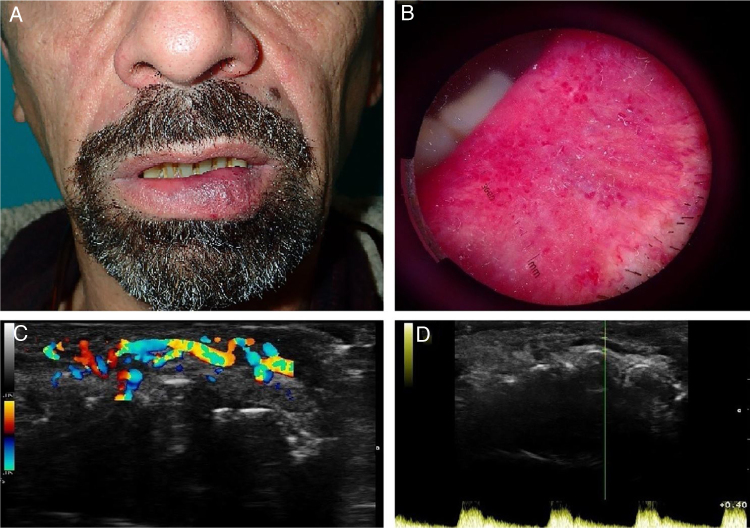
(a), Clinical photograph showing an erythematous-violaceous, non-ulcerated, soft-tissue tumoration on the left side of the lower lip. (b), Dermoscopy photograph of the lesion showing linear and tortuous blood vessels with superficial microulcerations. (c), A 15×4.7 mm thick submucosal artery with an underlying transverse channel is noted in this color Doppler image. (d), The pulsatile flow of the artery was clearly visible in the real-time color Doppler scan.

With the clinical suspicion of CPLA versus arteriovenous malformation, the authors made a color Doppler ultrasonography with a frequency range of 8–12 MHz that revealed a 15×4.7 mm thick submucosal artery with an underlying transverse channel. The pulsatile flow of the artery was clearly visible in the real-time color Doppler scan (Fig. 1).

### Case report 2

**Figure 2:**
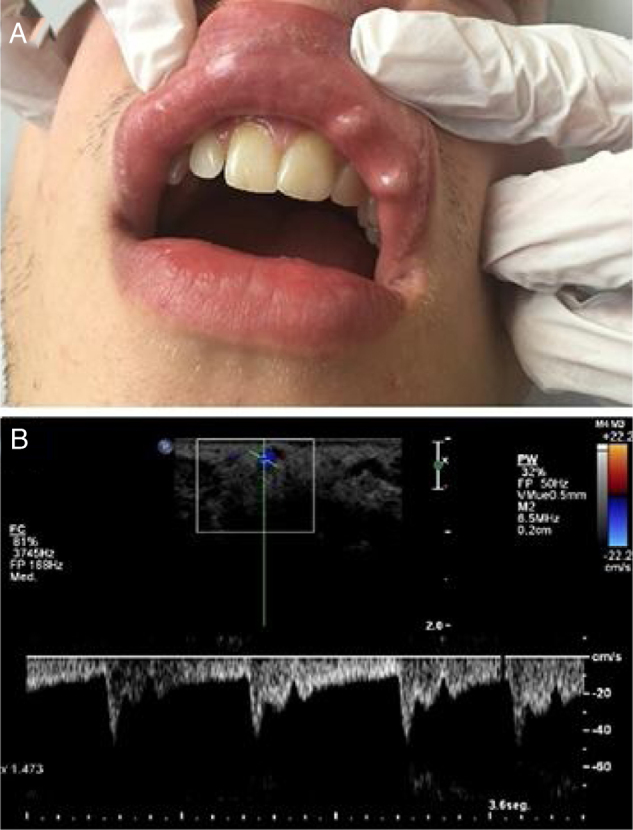
(a), Clinical photograph showing an enlarging reddish-blue, translucent, and pulsatile nodule of the right side of the lower lip of 7 mm. (b), Simultaneous spectral and color analysis using Doppler ultrasound show a high resistance forward flow in a large-diameter inferior labial artery branch at the lower lip.

A 25-year-old male with a nibbling habit had been aware of a slowly enlarging reddish-blue, translucent, and pulsatile nodule of the right side of his lower lip of 7 mm. He also refers history of recurrent microulcerations in lower lips for years. Color Doppler and spectral curve confirmed the suspicion of CPLA (Fig. 2).

### Case report 3

**Figure 3:**
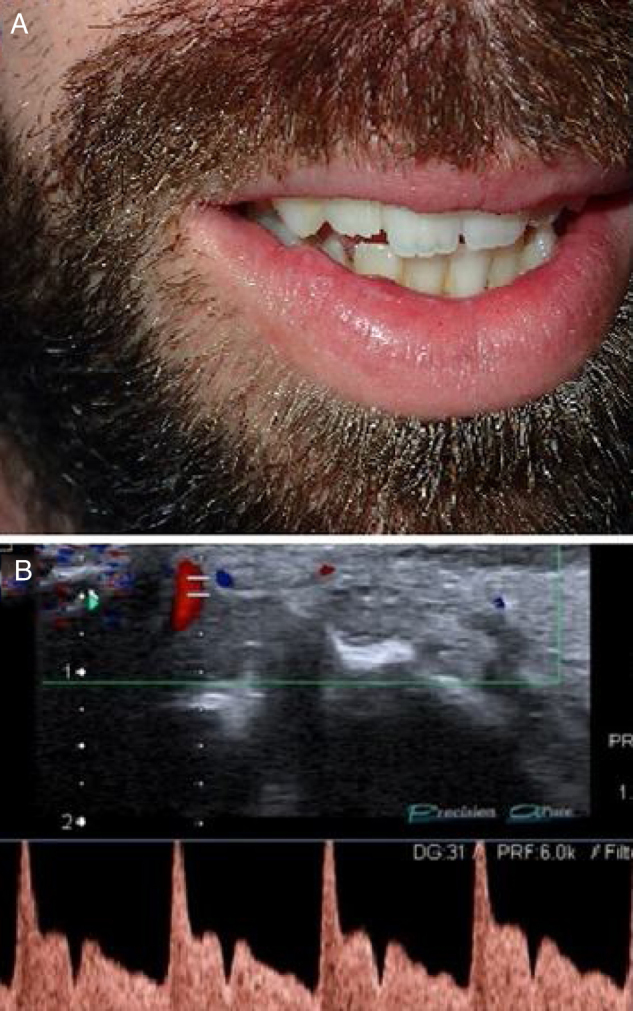
(a), Clinical photograph showing two asymptomatic, translucent, and pulsatile nodules of 1 cm each, in the left side of the upper lip. (b), Color Doppler ultrasound with spectral curve analysis confirms the arterial blood flow within the lesion.

A 24-year-old first noticed two asymptomatic, translucent, and pulsatile nodules of 1 cm each, in the left side of his upper lip. The clinical diagnosis was CPLA which was confirmed with color Doppler ultrasonography (Fig. 3).

## Discussion

CPLA is a vascular anomaly in which a primary arterial branch penetrates into the submucosal tissue without arborizing and without reduction in diameter.^1^

The incidence of CPLA is approximately 3%, with no differences between men and women, but it seems to be an underdiagnosed pathology. It occurs more frequently with aging, suggesting that a degenerative process of the vascular wall may be involved.^2^

Clinically, CPLA appears as a solitary, soft, elevated lesion of bluish color located close to the labial mucosa of the upper or lower lip, papular or linear, and visibly pulsatile. In some cases, the ischemia of subcutaneous cellular tissue which is linked with the diameter of the vessel and the depth of the artery to the mucosal surface could lead to chronic ulceration simulating a squamous cell carcinoma even no association has been demonstrated between them.^3^

Thus, the clinical diagnosis is easy if the lesion appears with these characteristics. The problem becomes when it appears as an asymptomatic, non-pulsatile and colorless papule forcing us to perform a differential diagnosis with other lesions as varicosities, haemangiomas, venous lakes, mucoceles, irritation fibroma, or vascular anomalies.^4^

An excisional biopsy of those lesions or even their surgical extirpation could have a fatal outcome with profuse bleeding.

As most CPLA lesions are benign and do not require treatment, except complications and/or patients demands, it is preferred to use the non-invasive diagnostic tool instead of invasive methods mainly high-resolution and color Doppler ultrasonography which allows direct visualization of the lesion, assessing its exact location and diameter at every axis, as well as the blood flow velocity.^5^

In conclusion, it is important for the clinicians to be aware of CPLA in daily clinical practice including it in the differential diagnosis of labial mucosal papules. Lateral pulsation in the papule is the distinctive sign of this pathology even the diagnosis should be confirmed with non-invasive diagnostic tools as Doppler ultrasonography relegating the biopsy to a second plane because of the risk of complications.

## Financial support

None declared.

## Authors’ contributions

José Antonio Llamas Carmona: Preparation and writing of the manuscript in consultation with the rest of the authors.

Ángela Rivera Mercado: Involved in study conception and planning.

Miguel Lova Navarro: Manuscript critical review.

Elisabeth Gómez Moyano: Of the final version of the manuscript.

## Conflicts of interest

None declared.
